# Juniperus communis extract induces cell cycle arrest and apoptosis of colorectal adenocarcinoma in vitro and in vivo

**DOI:** 10.1590/1414-431X2020e10891

**Published:** 2021-07-16

**Authors:** Wen-Lin Lai, Shan-Chih Lee, Kai-Fu Chang, Xiao-Fan Huang, Chia-Yu Li, Chien-Ju Lee, Chun-Yu Wu, Hui-Ju Hsu, Nu-Man Tsai

**Affiliations:** 1 Department of Medical Laboratory and Biotechnology, Chung Shan Medical University, Taichung, Republic of China, Taiwan; 2 Clinical Laboratory, Chung Shan Medical University Hospital, Taichung, Republic of China, Taiwan; 3 Department of Medical Imaging and Radiological Sciences, Chung Shan Medical University, Taichung, Taiwan, Republic of China; 4 Department of Medical Imaging, Chung Shan Medical University Hospital, Taichung, Taiwan, Republic of China; 5 Institute of Medicine, Chung Shan Medical University, Taichung, Taiwan, Republic of China; 6 Department of Life-and-Death Studies, Nanhua University, Chiayi, Taiwan, Republic of China; 7 Division of Cardiology, Department of Internal Medicine, Ditmanson Medical Foundation Chia-Yi Christian Hospital, Chia-Yi, Taiwan, Republic of China

**Keywords:** Colorectal cancer, Juniperus communis, Cell cycle, Apoptosis, Synergistic effect

## Abstract

*Juniperus communis* (JCo) is a well-known traditional Chinese medicinal plant that has been used to treat wounds, fever, swelling, and rheumatism. However, the mechanism underlying the anticancer effect of JCo extract on colorectal cancer (CRC) has not yet been elucidated. This study investigated the anticancer effects of JCo extract *in vitro* and *in vivo* as well as the precise molecular mechanisms. Cell viability was evaluated using the MTT assay. Cell cycle distribution was examined by flow cytometry analysis, and cell apoptosis was determined by the terminal deoxynucleotidyl transferase dUTP nick end labeling (TUNEL) assay. Protein expression was analyzed using western blotting. The *in vivo* activity of the JCo extract was evaluated using a xenograft BALB/c mouse model. The tumors and organs were examined through hematoxylin-eosin (HE) staining and immunohistochemistry. The results showed that JCo extract exhibited higher cytotoxicity against CRC cells than against normal cells and showed synergistic effects when combined with 5-fluorouracil. JCo extract induced cell cycle arrest at the G_0_/G_1_ phase via regulation of p53/p21 and CDK4/cyclin D1 and induced cell apoptosis via the extrinsic (FasL/Fas/caspase-8) and intrinsic (Bax/Bcl-2/caspase-9) apoptotic pathways. *In vivo* studies revealed that JCo extract suppressed tumor growth through the inhibition of proliferation and induction of apoptosis. In addition, there was no obvious change in body weight or histological morphology of normal organs after treatment. JCo extract suppressed CRC progression by inducing cell cycle arrest and apoptosis *in vitro* and *in vivo*, suggesting the potential application of JCo extract in the treatment of CRC.

## Introduction

Colorectal cancer (CRC) is the third most common malignant cancer in humans, accounting for nearly 9.2% of all annual cancer-related deaths worldwide (1). The tumorigenesis of CRC is multifactorial and associated with progressive accumulation of epigenetic and genetic alterations that result in the transformation of normal rectal mucosa into malignant metastatic carcinoma (2). Diet is one of the main environmental factors involved in the etiology of CRC, with about 90% of CRC cases related to high intakes of red meat, saturated fats, and n-6 polyunsaturated fatty acids and low intakes of vitamins and fibers (3). The standard treatment for CRC is surgery combined with radiotherapy or chemotherapy, depending on tumor size, location, and disease stage (4). However, the standard chemotherapy regimens, including 5-fluorouracil (5-FU), doxorubicin, and mitomycin, exhibit side effects including mucositis, diarrhea, and dermatitis (5–8). Therefore, there remains an unmet clinical requirement for novel anti-CRC agents or combination therapy for the treatment of CRC. Recently, natural materials have shown potential as preventive or therapeutic agents for various cancers (7,8).

Natural materials and natural health products have recently been used in the development of new drugs. The chemodrugs that are currently available are derived from natural materials such as plants, marine organisms, and microbes (9). Recent analyses have shown that at least 73 approved anticancer drugs in clinical use, including paclitaxel, vinblastine, topotecan, and etoposide, were derived from plants (10,11). Natural products have been widely used in the discovery of anticancer agents because of their diverse molecular structures and biological affinities (12). Natural products mainly include traditional and herbal medicines, and recent studies have focused on their biofunctions and applications in cancer therapy (13).

*Juniperus communis* (JCo) is an evergreen coniferous shrub belonging to the Cuppressaceae family (14). JCo extract has been traditionally used as an herbal medicine for the treatment of pain, wounds, fever, swelling, and rheumatism (15,16). Recent studies have demonstrated that JCo extract exhibits anti-oxidative, anti-microbial, anti-inflammatory, anti-diabetic, and anti-hyperlipidemic activities (17–20). The anticancer activity of JCo extract has been demonstrated based on tumor growth inhibition with regard to melanoma, oral cancer, breast cancer, lung cancer, and carcinomas of the liver and colon (21–24). The compounds in JCo extract, including α-pinene, d-limonene, and terpinolene, have shown anti-carcinogenic activities (25–31). However, few studies have investigated the anticancer mechanism of JCo extract *in vitro* and *in vivo*.

This study aimed to examine the anticancer effects of JCo extract *in vitro* and *in vivo* and to determine the precise molecular mechanism underlying the tumor growth inhibition induced by JCo extract in CRC. In addition, the combination of JCo extract and the clinical drug 5-FU was analyzed for its anticancer effects *in vitro* as well as the tolerance of JCo extract *in vivo*. Our results indicated the potential of JCo extract as a clinical therapeutic agent or adjuvant therapeutic agent in CRC therapy.

## Material and Methods

### Cell culture and reagents

Human colorectal adenocarcinoma (HT-29, ATCC^®^ HTB-38), mouse colon cancer (CT-26, BCRC 60447), canine kidney epithelial (MDCK, BCRC 60004), and mouse vascular endothelial (SVEC, BCRC 60220) cell lines were obtained from the American Type Culture Collection (ATCC, USA) or the Bioresource Collection and Research Center (BCRC, Taiwan). HT-29, SVEC, and MDCK cells were cultured in Dulbecco's modified Eagle’s medium (DMEM, Gibco BRL, USA); CT-26 cells were cultured in RPMI-1640 medium supplemented with 10% fetal bovine serum, HEPES (10 mM), pyruvate (1 mM), and penicillin/streptomycin solution (100 U/mL penicillin and 100 µg/mL streptomycin; all from Gibco BRL). The cells were routinely grown in culture dishes in a 5% CO_2_ humidified atmosphere at 37°C. The status of TP53 in HT-29 cells was determined by automated extraction of nucleic acids (AccuBioMed Co., Ltd., Taiwan) and sequencing using Femtopath Human Primer Sets (HongJing Biotech, Taiwan).

JCo fruits were freshly obtained from Nepal and subjected to extraction by steam distillation (22). The detailed extraction flowchart was tested on a small scale in our laboratory. Approximately 400 g of fruit was steam-distilled in a 2-L steam distillation unit for 100 min at 100-105°C at a flow rate of approximately 7.2 mL/min. Phoenix (USA) was then commissioned for the large-scale production of JCo extract. 5-FU and etoposide (VP-16) were purchased from Sigma-Aldrich (USA). Before each experiment, all extracts or chemicals were dissolved in dimethyl sulfoxide (DMSO, Sigma-Aldrich) and diluted in fresh medium.

### Determination of cytotoxicity

Cytotoxicity was analyzed using the 3-(4,5-dimethylthiazol-2-yl)-2,5-diphenyltetrazolium bromide (MTT) assay. Briefly, cells were cultured overnight in 96-well plates (5×10^3^ cells/well) containing the corresponding culture media, followed by treatment with various concentrations of JCo extract (0-100 μg/mL) for 24, 48, or 72 h. Next, the medium in each well was replaced with MTT solution (400 μg/mL; Sigma), and the plates were incubated for 6-8 h. After incubation, DMSO was added to solubilize the formazan crystals, and absorbance at 550 nm was measured using a microplate reader (Molecular Devices, Spec384, USA). The 50% inhibitory concentration (IC_50_) was calculated based on the graph of relative viability *vs* JCo extract concentration. Cell viability (%) was calculated as absorbance (treated cells) / absorbance (control cells) × 100. After testing the concentration of JCo extract (0-100 μg/mL) in HT-29 cells, the dose of 65 μg/mL (IC_70_), which induced cell death, was used in further experiments to study the anticancer mechanism against CRC cells.

### Synergistic effects of JCo extract and clinical drugs

HT-29 cells were treated with a combination of JCo extract (0, 20, 40, 60, and 80 μg/mL) and 0.25 μg/mL 5-FU, or a combination of 5-FU (0, 0.125, 0.25, 0.5, and 1 μg/mL) and 40 μg/mL JCo extract for 72 h. Cell viability was then determined by the MTT assay. The combination index (CI) was calculated as follows: [IC_50_ (drug A+B) / IC_50_ (drug A)] + [IC_50_ (drug A+B) / IC_50_ (drug B)]. The combination effects of drugs were evaluated based on the CIs for assessing synergism (CI<1), additivity (CI=1), and antagonism (CI>1) (32).

### Cell cycle analysis

Cell cycle distribution was determined using propidium iodide (PI) staining and flow cytometry analysis. Briefly, HT-29 cells (2×10^6^ cells/dish) were treated with 65 μg/mL JCo extract for 0, 6, 12, 24, and 48 h, followed by the addition of PI/RNase staining solution (40 μg/mL of PI and 100 μg/mL of RNase; Sigma) and incubation at 4°C overnight. The DNA content (FL2 intensity) of each cell was measured using FACScan (Beckton Dickinson, USA) and Kaluza Flow Cytometry Analysis software (version 1.2, Beckman Coulter, USA).

### Terminal deoxynucleotidyl transferase dUTP nick end labeling (TUNEL) assay

Apoptosis was detected using the *In Situ* Cell Death Detection kit, POD (Roche, Germany). Cells on silane-coated glass slides or deparaffinized tissue sections were rehydrated with phosphate-buffered saline (PBS), treated with 3% H_2_O_2_ in methanol to inactivate endogenous peroxidase, and incubated with permeabilization solution (0.1% Triton X-100 in 0.1% sodium citrate buffer) on ice. The samples were then incubated with the TUNEL reaction solution for 2 h at 37°C and counterstained with PI. TUNEL-positive cells were observed and photographed using a fluorescence microscope (ZEISS AXioskop2, Germany) at 400× magnification.

### Western blotting

JCo extract-treated cells were lysed with RIPA lysis buffer containing a protease inhibitor (Bio Basic Inc., Canada) and a phosphatase inhibitor (Bionovas, Canada) incubated on ice for 30 min. After centrifugation at 12,000 *g* for 30 min at 4°C, the protein content of the supernatant was measured using a bicinchoninic acid (BCA) protein assay kit (Pierce, USA). Equal amounts (20 μg) of protein samples were resolved using 8-12.5% sodium dodecyl sulfate-polyacrylamide gel electrophoresis (SDS-PAGE) and transferred to 0.22-μm polyvinylidene difluoride (PVDF) membranes (PALL Corp., USA). The membranes were blocked with 5% nonfat dry milk and incubated with the primary antibodies anti-p53, anti-p-p53, anti-Rb, anti-pRb, anti-CDK2, anti-CDK4, anti-cyclin A, anti-cyclin B1, anti-cyclin D1, anti-FasL, anti-Fas, anti-Bax, anti-Bcl-2, anti-caspase-8, anti-caspase-9, anti-caspase-3, anti-proliferating cell nuclear antigen (PCNA), anti-vascular endothelial growth factor (VEGF), anti-VEGF receptor 1 (VEGFR1), anti-VEGF receptor 2 (VEGFR2), anti-matrix metalloproteinase (MMP)-2, anti-MMP-9 (Santa Cruz, USA), anti-p21, and anti-β-actin (iReal Biotechnology Co., Ltd., Taiwan) overnight at 4°C. The membranes were washed three times with 0.5% Tween-20 in tris-buffered saline and incubated with biotin-conjugated secondary antibodies (Santa Cruz) for 2 h, followed by incubation with peroxidase-conjugated streptavidin (Jackson ImmunoResearch Inc., USA) for 1 h. Antibody-reactive proteins were treated with an enhanced chemiluminescence reagent (ECL, T-Pro Biotechnology, Taiwan) and detected using a fluorescence/chemiluminescence imaging analyzer (GE LAS-4000, GE Healthcare Life Sciences, USA). Protein expression was quantified using ImageJ 1.47t software (NIH, USA) and calculated as follows: (sample intensity / β-actin intensity of the sample) / (control intensity / β-actin intensity of the control).

### Detection of caspase-3 activation

HT-29 cells (5×10^5^ cells/well in a 6-well culture plate) were pretreated with caspase-3 inhibitor (1 μM; Z-DEVD-FMK, BIOSCIENCES, USA) for 2 h, followed by treatment with 65 μg/mL JCo extract for 24 h. The expression levels of pro-caspase-3 and cleaved caspase-3 in treated cells were determined by western blotting.

### *In vivo* tumor growth and immunohistochemistry

Female BALB/c mice (10-12 weeks; 19-23 g) were obtained from the National Laboratory Animal Center (Taiwan), housed (6 per cage) in a laminar airflow room, maintained with a 12-h light/dark cycle (relative humidity: 55-60%; temperature: 25±1°C), and allowed free access to a balanced diet and water. Before experimentation, the mice were acclimated to laboratory conditions for 1 week. The experiment was performed at Chung Shan Medical University (CSMU) according to the Guide for the Care and Use of Laboratory Animals. The CRC model was established by injecting 1×10^6^ CT-26 cells *sc* into the flanks of BALB/c mice. The mice were randomized into the vehicle control (n=4) and JCo extract treatment (n=6) groups. When the tumor volumes exceeded 15 mm^3^ (cell injection for 7 days), the tumor-bearing mice were treated with 200 mg/kg JCo extract once every 2 days for 40 days and sacrificed by carbon dioxide asphyxiation when the tumor volume exceeded 1500 mm^3^ (L×H×W×π/6 mm^3^). This procedure was approved by the Institutional Animal Care and Use Committee (IACUC) of CSMU (allowance number: CSMU-IACUC-1543). The tumor masses and organs were collected, fixed with 4% neutral formalin, embedded in paraffin, and cut into sections for immunohistochemical (IHC) and hematoxylin-eosin (HE) staining.

The sections were deparaffinized and rehydrated, and the endogenous peroxidase was inactivated. The sections were blocked with 10% bovine serum albumin (BSA) in PBS and incubated at 4°C overnight with the primary antibodies anti-PCNA, anti-VEGF, anti-VEGFR1, anti-VEGFR2, anti-MMP-2, anti-MMP-9, and anti-cleaved caspase-3 (Santa Cruz). After washing, the slides were incubated with a biotinylated secondary antibody (Super Sensitive Polymer HRP IHC Detection System kit, BioGenex, USA) for 2 h. Finally, the slides were incubated with an avidin-biotin complex, reacted with 3,3′-diaminobenzidine (DAB), and counterstained with hematoxylin. All samples were observed and photographed using a microscope and scored using the Quickscore method (33).

### Statistical analyses

The data are reported as means±SD or standard error. The IC_50_ values were determined by linear regression analysis using Microsoft Excel 2016 (USA). Statistical significance was determined using the Student's *t*-test and one-way analysis of variance. Survival analyses were performed using the Kaplan-Meier method. P-values <0.05 were considered statistically significant.

## Results

### JCo extract decreased the viability of CRC cells

**Figure 1:**
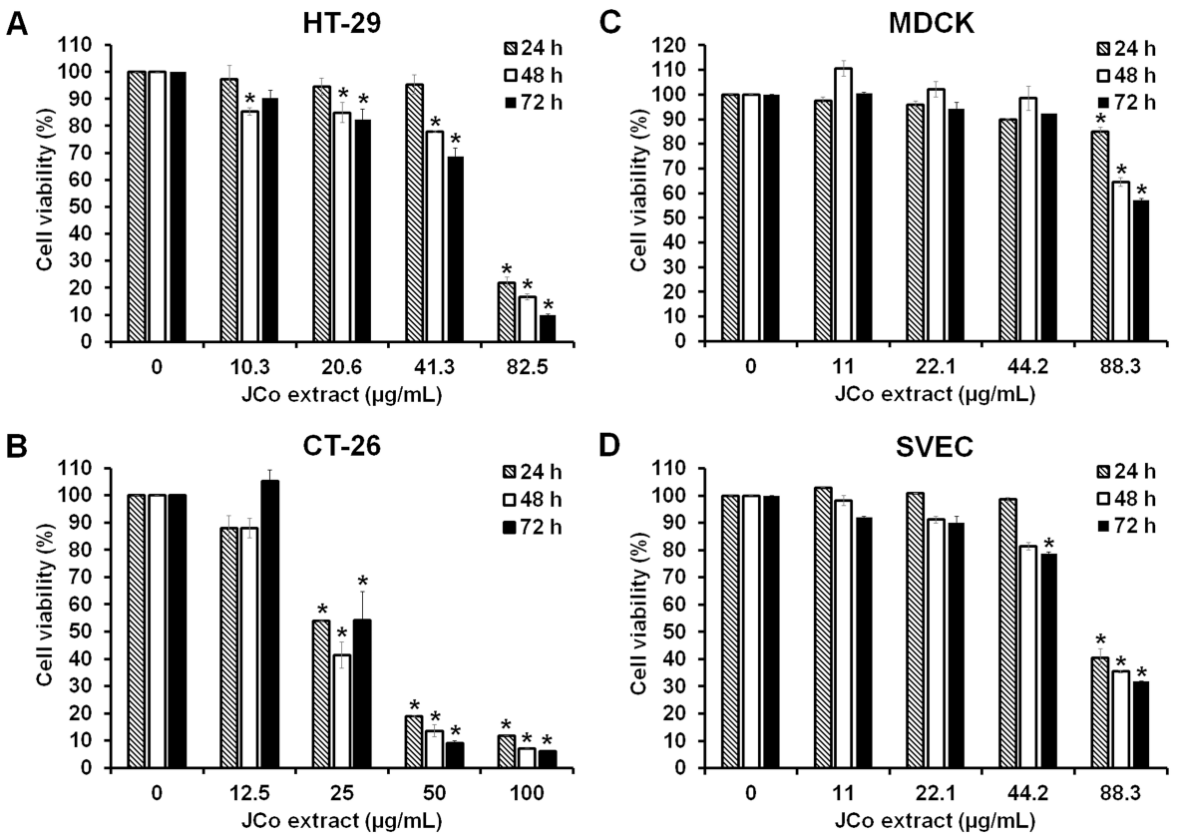
Effect of *Juniperus communis* (JCo) extract on growth inhibition in colorectal cancer cell lines. HT-29 (**A**), CT-26 (**B**), MDCK (**C**), and SVEC (**D**) cells were treated with serial dilutions of JCo extract for 24, 48, and 72 h, and cell viability was evaluated by the 3-(4,5-dimethylthiazol-2-yl)-2,5-diphenyltetrazolium bromide (MTT) assay. Data are reported as means±SD. *P<0.05 *vs* control (ANOVA).

CRC cells were treated with various concentrations of JCo extract, and cell viability was detected by the MTT assay. The results showed that JCo extract reduced the viability of HT-29 and CT-26 cells in a dose-dependent manner (Figure 1). As shown in Table 1, the mean IC_50_ of JCo extract in tumor cells (HT-29 and CT-26 cells) was significantly lower than that in normal cells (MDCK and SVEC cells), indicating higher selectivity of JCo extract for tumor cells than for normal cells.

**Table 1:** Cytotoxicity (IC_50_) of *Juniperus communis* (JCo) extract in colorectal cancer cells and normal cells.

Drugs	Colorectal cancer cells		Normal cells	
	HT-29	CT-26	MDCK	SVEC
JCo extract				
24 h	66.71±0.48.0^a,c^	27.8±0.2^a,b,c^	>88.3^c^	81.1±2.2^b,c^
48 h	60.02±0.21^a,b,c^	22.7±0.9^a,b,c^	>88.3^c^	74.8±0.3^b,c^
72 h	54.32±1.58^a,b,c^	27.3±4.5^a,b,c^	>88.3^b,c^	71.2±0.6^b,c^
5-FU				
24 h	>10	2.7±0.03^a^	>10	6.8±1.3
48 h	1.3±0.4	0.3±0.02^a^	>10	1.4±0.05
72 h	0.7±0.03	0.4±0.01^a^	1.1±0.01	0.8±0.03
VP-16				
24 h	>100^a^	44.1±0.4	66.0±1.0	46.2±3.2
48 h	3.3±2.2	9.4±0.03	25.7±2.7	2.2±0.6
72 h	2.3±0.02	4.1±0.1^a^	2.7±0.2	1.0±0.001

IC_50_: half maximal inhibitory concentration. Data are reported as means±SD in µg/mL. ^a^P<0.05 between tumor cells and normal cells; ^b^P<0.05 between JCo extract group and 5-FU treatment group; ^c^P<0.05 between JCo extract group and VP-16 treatment group (ANOVA).

### Synergistic effects of JCo extract and 5-FU

**Figure 2:**
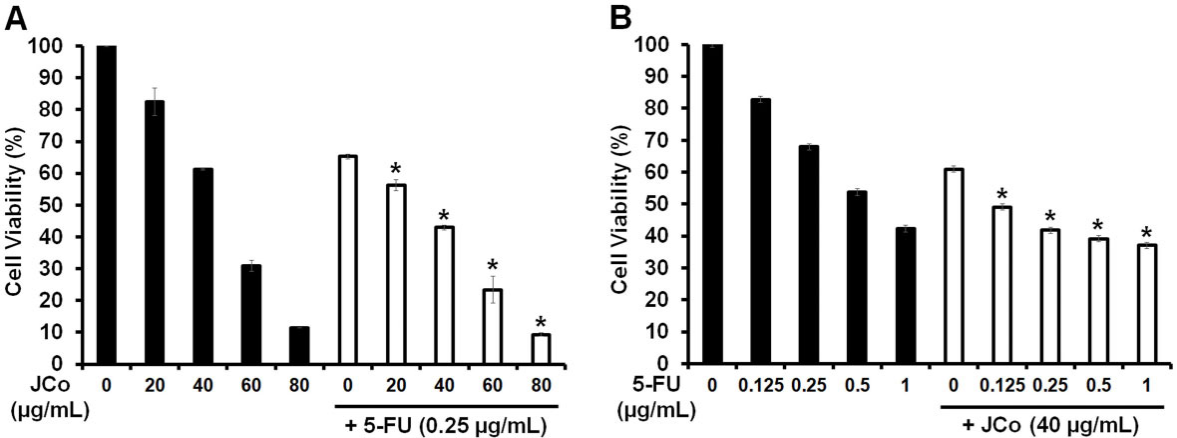
*Juniperus communis* (JCo) extract enhances the sensitivity of colorectal cancer cells to 5-fluorouracil (5-FU). HT-29 cells were treated with (**A**) JCo extract (0, 20, 40, 60, and 80 μg/mL) alone or JCo extract in combination with 0.25 μg/mL 5-FU or with (**B**) 5-FU (0, 0.125, 0.25, 0.5, and 1 μg/mL) alone or 5-FU in combination with 40 μg/mL JCo extract for 72 h. Cell viability was evaluated by the 3-(4,5-dimethylthiazol-2-yl)-2,5-diphenyltetrazolium bromide (MTT) assay. Data are reported as means±SD. *P*<*0.05 *vs* single drug in the combination group. JCo extract in combination with 5-FU displayed a synergistic effect (CI<1) (ANOVA).

To analyze whether JCo extract had a synergistic, additive, or antagonistic effect when administered in combination with 5-FU, HT-29 cells were treated with JCo extract (0-80 μg/mL) combined with 0.25 μg/mL 5-FU or with 5-FU (0-1 μg/mL) combined with 40 μg/mL JCo extract. After 72 h of treatment, cell viability was determined by the MTT assay. As shown in Figure 2, the viability of CRC cells was lower after treatment with JCo extract combined with 0.25 μg/mL 5-FU (42.94±0.69%) than that after treatment with JCo extract alone (61.30±0.17%). In contrast, cell viability decreased after treatment with 5-FU combined with 40 μg/mL JCo extract (41.82±1.45%) compared to that after treatment with 5-FU alone (67.94±0.35%). The synergistic, additive, or antagonistic effects of the drug combinations were determined using the Chou-Talalay method (32). The CI was 0.79, suggesting a synergistic effect of the combination of JCo extract and 5-FU (CI<1) after a treatment period of 72 h.

### JCo extract induced cell cycle arrest at the G_0_/G_1_ phase in CRC cells

**Figure 3:**
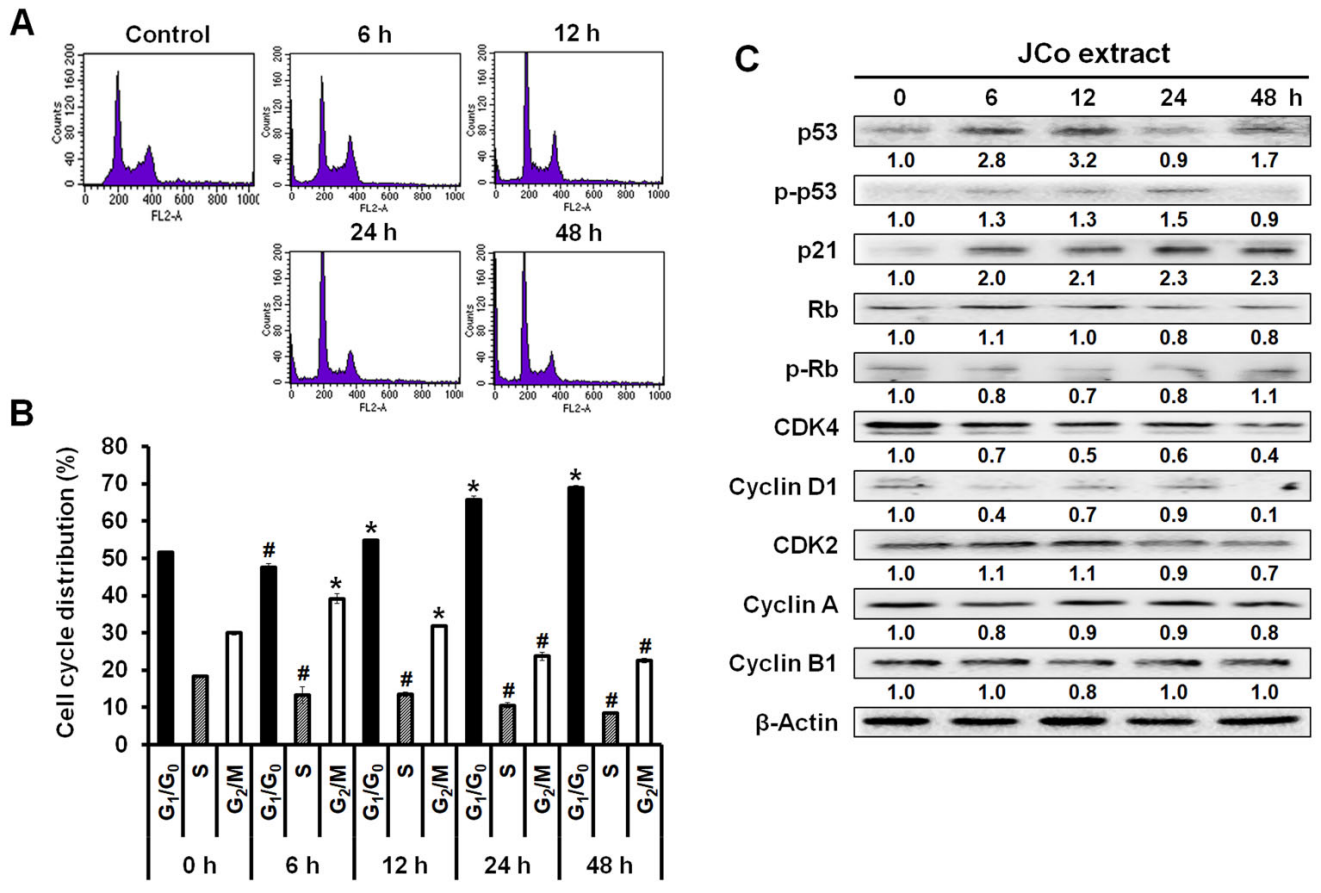
*Juniperus communis* (JCo) extract induces G_0_/G_1_ arrest and regulates the expression of cell cycle-associated proteins in HT-29 cells. **A**, HT-29 cells were treated with 65 μg/mL JCo extract for 0, 6, 12, 24, and 48 h, stained with propidium iodide, and analyzed for FL2 intensity by flow cytometry. **B**, Cell cycle distribution (G_0_/G_1_, S, and G_2_/M phases) in JCo extract-treated cells was analyzed using Kaluza Flow Cytometry Analysis software. Data are reported as means±SD. *P<0.05 *vs* control with a significant increase, ^#^P<0.05 *vs* control with a significant decrease (ANOVA). **C**, The expression of cell cycle-associated proteins in JCo extract-treated cells was determined by western blotting.

CRC cells treated with JCo extract were collected and stained with PI. Cell cycle distribution was then analyzed by measuring FL2 intensity using a flow cytometer (Figure 3A). The results showed that the percentage of G_0_/G_1_ phase cells increased from 51.66±0.19% to 68.86±0.47% within 48 h of JCo extract treatment, while the percentages of S and G_2_/M phase cells decreased from 18.34±0.2 to 8.48±0.07% and 29.99±0.48 to 22.66±0.66%, respectively (Figure 3B).

In addition, JCo extract regulated the expression of proteins related to the cell cycle, including cell cycle regulators (p53, p-p53, and p21), tumor suppressors (Rb and p-Rb), and regulatory molecules involved in the G_0_/G_1_ phase (CDK4 and cyclin D1, Figure 3C). Taken together, these results suggested that the tumor growth inhibition effect of JCo extract was associated with the induction of cell cycle arrest via the regulation of cell cycle-related protein expression.

### JCo extract triggered cell apoptosis by regulating the caspase cascade

**Figure 4:**
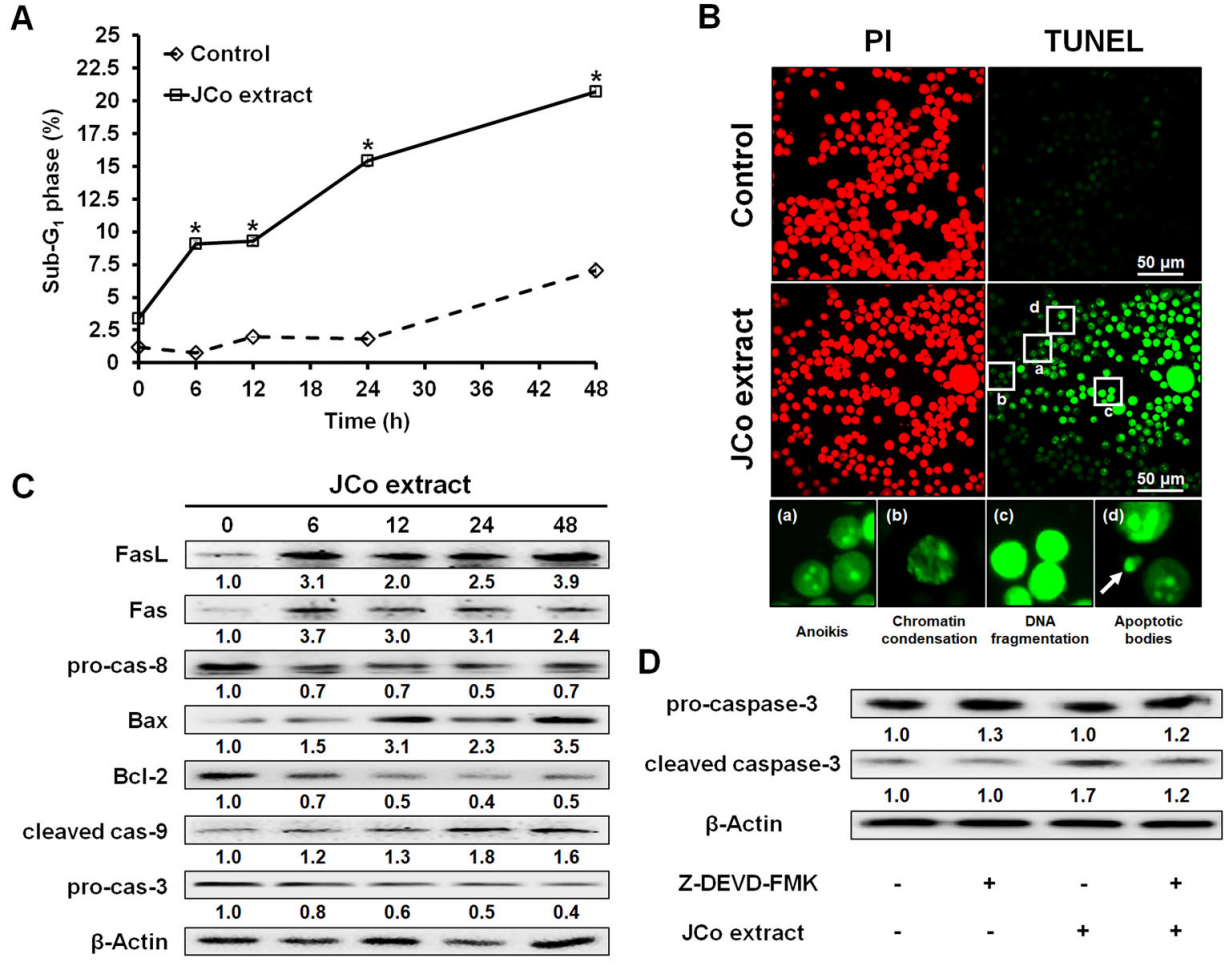
Effects of *Juniperus communis* (JCo) extract on the extrinsic and intrinsic apoptotic pathways in HT-29 cells. **A**, The percentage of SubG_1_ phase cells after JCo extract treatment was analyzed by flow cytometry. Data are reported as means±SD. *P<0.05 *vs* control (ANOVA). **B**, Cell apoptosis was determined after treatment with 65 μg/mL JCo extract for 48 h by TUNEL assay (scale bar 50 μm). The apoptotic morphologies included anoikis, chromatin condensation, DNA fragmentation, and the appearance of apoptotic bodies (arrow). **C**, The protein expression levels of the components of the extrinsic and intrinsic apoptotic pathways in JCo extract-treated cells were analyzed by western blotting. **D**, HT-29 cells pretreated with 1 μM Z-DEVD-FMK (caspase-3 inhibitor) for 2 h were treated with 65 μg/mL JCo extract for 24 h, and caspase-3 activation was determined by western blotting.

The percentage of cells in the SubG_1_ phase (apoptosis peak) significantly increased from 5.61±0.19 to 20.70±0.09% after treatment with JCo extract (Figure 4A). To determine whether this increase was due to the induction of cell apoptosis by JCo extract, we used the TUNEL assay to detect apoptosis in JCo extract-treated cells. The data revealed that JCo extract-treated cells were TUNEL-positive and showed apoptotic morphologies, including anoikis, chromatin condensation, and DNA fragmentation, as well as the presence of apoptotic bodies (Figure 4B).

Next, to identify the apoptotic pathway activated by treatment with JCo extract in HT-29 cells, we performed western blotting. We found that JCo extract activated both the extrinsic (FasL, Fas, and pro-caspase-8) and intrinsic (Bax, Bcl-2, and cleaved caspase-9) apoptotic pathways (Figure 4C). Furthermore, JCo extract-induced activation of caspase-3 was blocked by the pretreatment of cells with a caspase-3 inhibitor (Figure 4D). Together, these results indicated that JCo extract activated the extrinsic and intrinsic pathways of apoptosis to trigger apoptosis and tumor cell death.

### JCo extract downregulated the expression of angiogenesis- and metastasis-associated proteins

**Figure 5:**
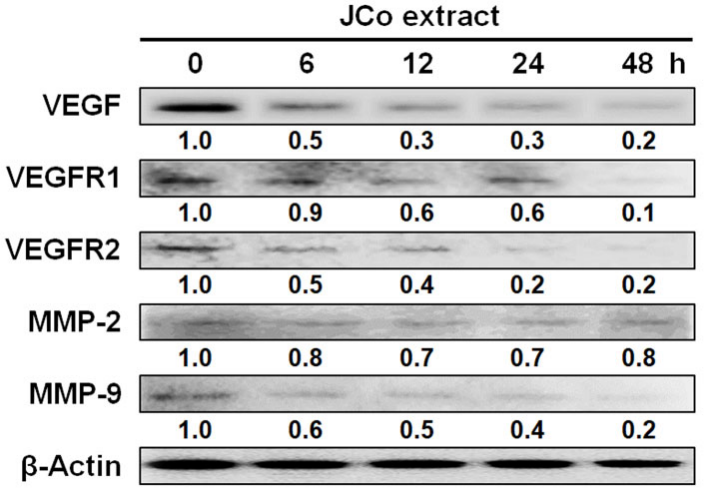
Expression of proteins involved in autocrine angiogenesis and metastasis in *Juniperus communis* (JCo) extract-treated HT-29 cells. HT-29 cells were treated with 65 μg/mL JCo extract for 0, 6, 12, 24, and 48 h, and the expressions of vascular endothelial growth factor (VEGF), VEGF receptor 1 (VEGFR1), VEGF receptor 2 (VEGFR2), matrix metalloproteinase (MMP)-2, and MMP-9 were analyzed by western blotting.

The effects of JCo extract on angiogenesis and metastasis were determined by western blotting. The results showed that JCo extract treatment decreased the protein expression levels of the autocrine angiogenesis-associated proteins VEGF, VEGFR1, and VEGFR2 and the metastasis-associated protein MMP-9 (Figure 5). Therefore, JCo extract may inhibit tumor proliferation, angiogenesis, and metastasis by downregulating the expression of these associated proteins.

### Effects of JCo extract on CRC in tumor-bearing mice

**Figure 6:**
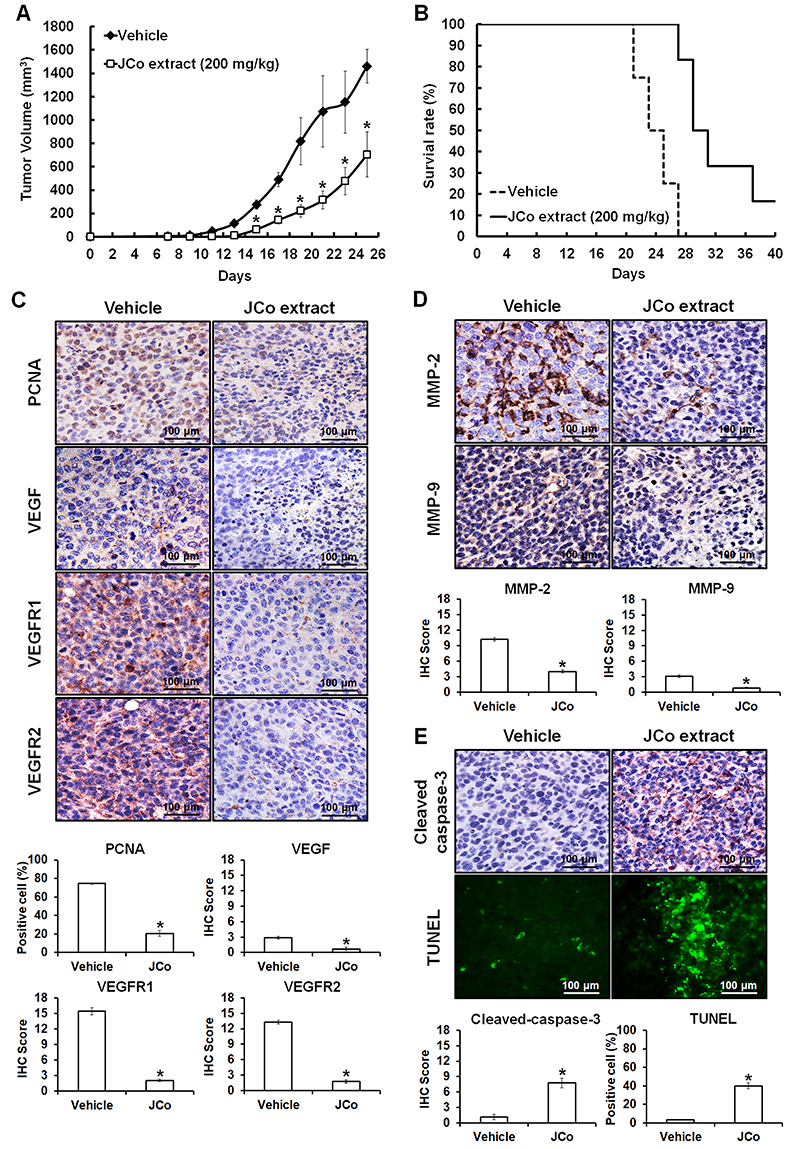
Effect of *Juniperus communis* (JCo) extract on the inhibition of CT-26 tumors in a BALB/c mouse model. Subcutaneous tumor-bearing mice were treated with 200 mg/kg JCo extract (*sc*) once every 2 days for 40 days. **A** and **B**, Tumor volumes were calculated once every 2 days, and the mice were sacrificed when the tumor volume exceeded 1500 mm^3^. **C**-**E**, Protein expression levels of proliferating cell nuclear antigen (PCNA), vascular endothelial growth factor (VEGF), VEGF receptor 1 (VEGFR1), VEGF receptor 2 (VEGFR2), matrix metalloproteinase (MMP)-2, MMP-9, and cleaved caspase-3 were detected by immunohistochemistry and scored using the Quickscore method (scale bars 100 μm). JCo extract-induced cell apoptosis was measured using the TUNEL assay. Data are reported as means±SD. *P<0.05 *vs* vehicle (ANOVA or *t*-test).

To analyze tumor growth suppression *in vivo*, CRC mouse models bearing tumors were established. The results showed a significantly lower tumor volume in mice treated with JCo extract (703±192.80 mm^3^) than that in the vehicle group (1459.84±144.14 mm^3^) at day 25 (Figure 6A). The survival rate was 100% in the JCo extract treatment group but only 25% in the vehicle group (Figure 6B). Thus, JCo extract suppressed tumor growth and prolonged life expectancy in animals.

Next, we investigated the effects of JCo extract on major regulatory proteins, including cell proliferation markers (PCNA), angiogenesis proteins (VEGF/VEGFR1/VEGFR2), metastasis proteins (MMP-2/MMP-9), and apoptosis proteins (cleaved caspase-3), *in vivo* by IHC analysis. The results showed that JCo extract decreased the protein expression levels of PCNA (27.6%), VEGF (24.1%), VEGFR1 (13.3%), VEGFR2 (13.4%), MMP-2 (39.1%), and MMP-9 (26.4%) and increased the expression of cleaved caspase-3 (692.1%) compared to those in the vehicle group (values in the vehicle group were regarded as 100%) (Figure 6C-E). The TUNEL positivity rate was 3.2±0.33% in the vehicle group and 40±3.33% in the JCo treatment group, indicating that JCo extract triggered increased apoptosis (Figure 6E). These results suggested that JCo extract suppressed tumor development through the inhibition of tumor proliferation and induction of cell apoptosis.

**Figure 7:**
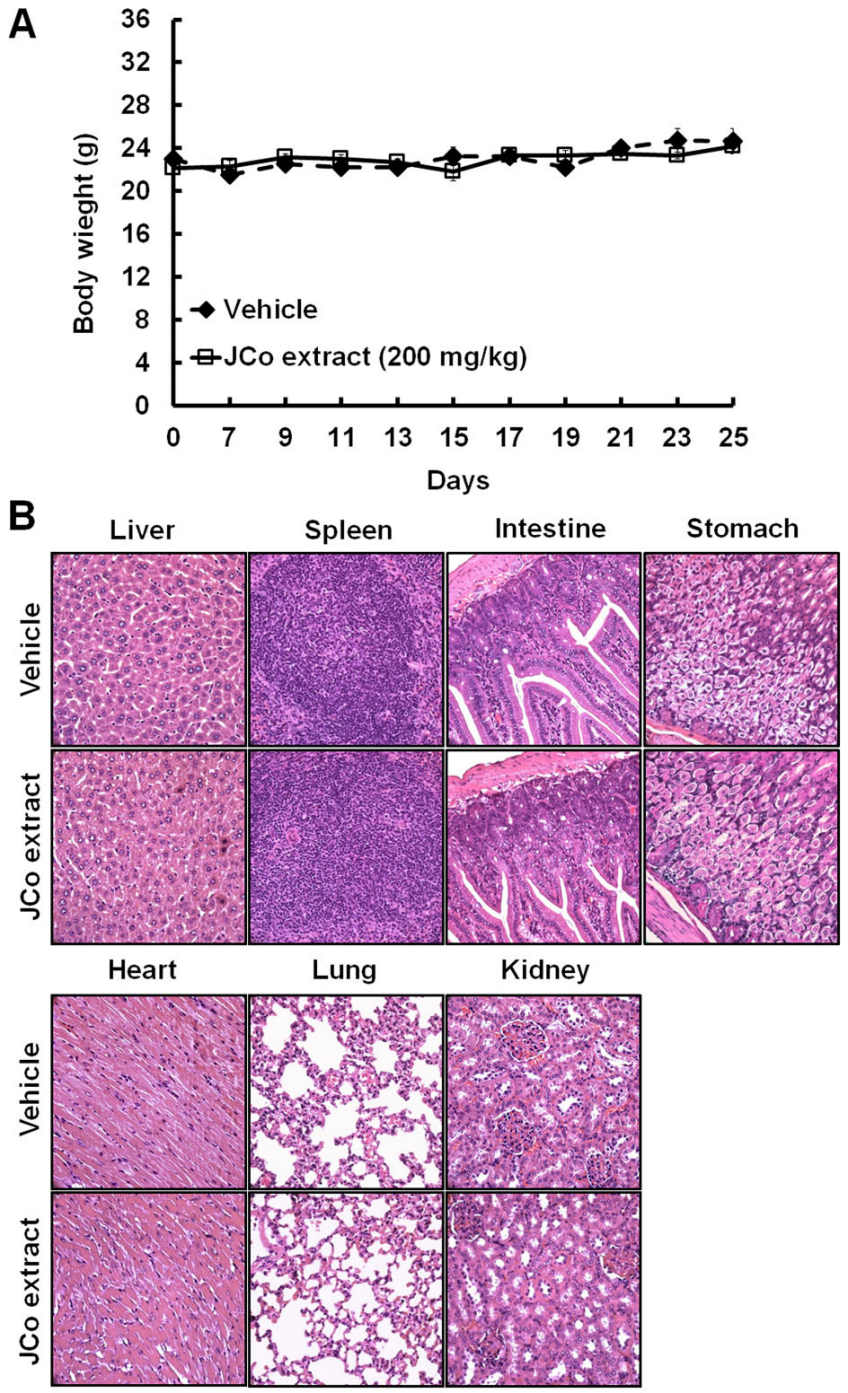
Effects of *Juniperus communis* (JCo) extract on mouse body weight and vital organs. **A**, The body weights of tumor-bearing mice were recorded once every 2 days after JCo extract treatment for 40 days. Data are reported as means±SD. **B**, The vital organs were collected and analyzed by hematoxylin-eosin (HE) staining. No significant differences between the JCo extract and vehicle groups were noted in terms of body weight and histological morphology (scale bars, 100 μm).

In animal studies, no obvious loss of body weight or damage to the liver, spleen, intestine, stomach, heart, lung, and kidney were observed in the JCo extract treatment group compared to that in the vehicle group (Figure 7A and B), suggesting that the therapeutic course of JCo extract was well tolerated.

## Discussion

Accumulating evidence suggests that many herbal extracts and mixtures have anticancer and chemopreventive effects based on the disruption of the cell cycle and induction of apoptosis (34,35). JCo is a well-known plant with a long history of use in traditional Chinese medicine and herbal medicine. While JCo has shown anticancer effects against lung cancer, breast cancer, neuroblastoma, liver cancer, and colon cancer, the molecular mechanisms underlying these effects are not yet clearly understood. Our results demonstrated that JCo extract inhibited tumor cell growth *in vitro* and *in vivo* and enhanced the survival rate of tumor-bearing mice. In addition, JCo extract combined with 5-FU had a synergistic effect in CRC cells. In the context of molecular mechanisms, our results showed that JCo extract induced cell cycle arrest and activated the extrinsic and intrinsic apoptotic pathways to trigger tumor cell death. Furthermore, JCo extract treatment decreased the expression of proteins associated with autocrine angiogenesis and metastasis. A similar mechanism of action was detected in the animal model using IHC staining. Importantly, JCo extract exhibited lower cytotoxicity against normal cells and little or no organ damage in mice treated with JCo extract for 40 days.

The cell cycle plays an important role in the regulation of cell proliferation, division, and growth and is a target of many cancer therapeutic drugs (36). A recent study reported that the treatment of A549, MCF7, TK6, and U937 human cell lines with JCo extract (made from branches and leaves) resulted in the enhanced accumulation of G_2_/M phase cells (23). Another study showed that treatment with JCo extract (made from berries) increased the percentages of cells in the G_2_, M, and G_0_ phases and led to cell death in liver and colon carcinomas and myosarcoma (24). In our study, treatment with JCo extract upregulated p53 protein expression and downregulated p21 protein expression. p21 is a cyclin-dependent kinase inhibitor that regulates different phases of the cell cycle. The protein expression levels of CDK4/cyclin D1, which regulate the G_1_/S transition, were reduced after treatment with JCo extract, which triggered the accumulation of G_0_/G_1_ phase cells.

Apoptosis, a form of programmed cell death, is an important physiological process that balances cell formation and cell death without inducing an inflammatory response (37). Moreover, the induction of apoptosis is an important therapeutic strategy in cancer treatment. Apoptosis is mediated by two major pathways, the extrinsic (death receptor) and intrinsic (mitochondrial disruption) apoptotic pathways, which involve the activation of caspase-8 and caspase-9, respectively, to trigger caspase-3 activation (30,35). Therefore, we performed western blotting to identify the apoptotic pathway activated by JCo extract in CRC cells. Our results revealed that both the extrinsic (FasL, Fas, and pro-caspase-8) and intrinsic (Bax, Bcl-2, and cleaved caspase-9) apoptotic pathways were activated by JCo extract, finally resulting in apoptosis and morphological changes, including anoikis, chromatin condensation, DNA fragmentation, and the appearance of apoptotic bodies.

To investigate the anticancer effect of JCo extract and the underlying mechanism *in vivo*, a CT-26 tumor-bearing mouse model was established. The mean tumor volume was significantly reduced and the survival rate was significantly increased in the JCo extract treatment group compared to those in the vehicle group. IHC staining showed that JCo extract inhibited cell proliferation, autocrine angiogenesis, and metastasis and induced apoptosis in tumor-bearing mice; these findings are consistent with those obtained in the *in vitro* assays. Moreover, there was no significant difference between the JCo extract treatment and vehicle groups in terms of body weight and the histological morphology of the liver, spleen, intestine, stomach, heart, lungs, and kidneys after 40 days of treatment. These results indicated that JCo extract exhibited anti-proliferative, anti-angiogenic, and anti-metastatic activities and triggered apoptosis in CRC cells both *in vitro* and *in vivo*, without displaying obvious cytotoxicity against normal cells or organs at a low and well-tolerated dose.

Previous studies have demonstrated that JCo contains many pure natural compounds with anticancer effects. For example, deoxypodophyllotoxin isolated from JCo is a potent inducer of caspase-dependent apoptosis mediated by the mitochondrial (intrinsic) pathway and also inhibits cell survival via the MAPK/ERK and NFκB signaling pathways in malignant breast cancer cells (38). Another study showed that podophyllotoxin and deoxypodophyllotoxin isolated from selected *Juniperus* species are effective against leukemia cell lines (39). Imbricatolic acid isolated from the methanolic extract of JCo (made from berries) induces the accumulation of G_1_ phase cells and the downregulation of cyclins A, D1, and E1 in CaLu-6 cells (40). In this study, the major components of JCo extract with molecular weights less than 500 Da included α-pinene (27.8%), carane (14.3%), d-limonene (10.7%), and terpinolene (7.4%), as determined using gas chromatography-mass spectrometry (GC-MS) and the National Institute of Standards and Technology and Wiley library databases. Previous studies have shown that α-pinene induces cell cycle arrest in the G_2_/M phase in human hepatoma cell lines (25), has a synergistic effect against non-small-cell lung carcinoma when combined with paclitaxel (26), and induces apoptosis and confers anti-metastatic protection with respect to melanoma (27). d-limonene induces autophagy in SH-SY5Y neuroblastoma cells (28), inhibits angiogenesis and metastasis, induces cell death in human colon cancer cells (29), and induces cell apoptosis via a caspase-dependent mitochondrial pathway in human leukemia cells (30). Terpinolene, a common component of plants such as sage and rosemary, inhibits cell proliferation by decreasing the protein expression level of AKT1 in K562 cells (31). The results of these studies indicate that JCo extract contains anticancer compounds and that this extract shows potential as a future anticancer agent or an adjuvant therapeutic agent in CRC therapy.

In conclusion, our results demonstrated that JCo extract suppressed CRC cell growth both *in vitro* and *in vivo* and also showed a synergistic effect in combination with 5-FU. Therefore, JCo extract may serve as a potential therapeutic agent for the treatment of CRC.
